# Evolutionary relationships, hybridization and diversification under domestication of the locoto chile (*Capsicum pubescens*) and its wild relatives

**DOI:** 10.3389/fpls.2024.1353991

**Published:** 2024-02-23

**Authors:** Nahuel E. Palombo, Hanna Weiss-Schneeweiss, Carolina Carrizo García

**Affiliations:** ^1^ Instituto Multidisciplinario de Biología Vegetal, Universidad Nacional de Córdoba, CONICET, Córdoba, Argentina; ^2^ Department of Botany and Biodiversity Research, University of Vienna, Vienna, Austria

**Keywords:** Andean chiles, *Capsicum pubescens* gene pool, domestication, crop wild relatives, hybrids, RADseq

## Abstract

Patterns of genetic variation in crops are the result of multiple processes that have occurred during their domestication and improvement, and are influenced by their wild progenitors that often remain understudied. The locoto chile, *Capsicum pubescens*, is a crop grown mainly in mid-highlands of South-Central America. This species is not known from the wild and exists only as a cultigen. The evolutionary affinities and exact origin of *C. pubescens* have still not been elucidated, with hypotheses suggesting its genetic relatedness and origin to two wild putative ancestral *Capsicum* species from the Central Andes, *C. eximium* and *C. cardenasii*. In the current study, RAD-sequencing was applied to obtain genome-wide data for 48 individuals of *C. pubescens* and its wild allies representing different geographical areas. Bayesian, Maximum Likelihood and coalescent-based analytical approaches were used to reconstruct population genetic patterns and phylogenetic relationships of the studied species. The results revealed that *C. pubescens* forms a well-defined monotypic lineage closely related to wild *C. cardenasii* and *C. eximium*, and also to *C. eshbaughii*. The primary lineages associated with the diversification under domestication of *C. pubescens* were also identified. Although direct ancestor-descendant relationship could not be inferred within this group of taxa, hybridization events were detected between *C. pubescens* and both *C. cardenasii* and *C. eximium*. Therefore, although hybrid origin of *C. pubescens* could not be inferred, gene flow involving its wild siblings was shown to be an important factor contributing to its contemporary genetic diversity. The data allowed for the inference of the center of origin of *C. pubescens* in central-western Bolivia highlands and for better understanding of the dynamics of its gene pool. The results of this study are essential for germplasm conservation and breeding purposes, and provide excellent basis for further research of the locoto chile and its wild relatives.

## 1 Introduction

Patterns of genetic variation in cultivated plants result from multiple evolutionary processes. To understand these patterns and processes, phylogenetic reconstructions within the context of the putative ancestral wild relatives are essential. Such species-wide assessments often allow for identification of the ancestral lineages that gave rise to early domesticates and modern cultivars, and may provide insights into the factors contributing to the observed distribution of genetic diversity across gene pools (Glaszmann et al., 2010; Meyer et al., 2012). This also applies to domesticated species with unknown wild forms, as the related wild species may represent an extended gene pool (Brozynska et al., 2016; Bohra et al., 2022). Hybridization and introgression between cultivated forms and wild relatives contribute significantly to the formation and evolution of domesticated species. Ongoing natural and artificial introgression is a major factor shaping the current genetic diversity of modern crops (Jarvis and Hodgkin, 1999; Ellstrand et al., 2013). Therefore, better understanding of the origin of crops in the context of their wild relatives is crucial for developing strategies for the conservation and sustainable use of their diversity (Mastretta-Yanes et al., 2018; Pironon et al., 2020), especially considering crop genetic erosion due to global climate change and biodiversity loss (Khoury et al., 2022).

*Capsicum pubescens* Ruiz & Pav. (Solanaceae), commonly known as ‘locoto’ or ‘rocoto’, is a chile pepper species with a major cultural and economic importance in the Central Andes (i.e., Bolivia, Peru and Ecuador). The species is cultivated mainly in mid- and highlands from north-western Argentina to central Mexico (Heiser and Smith, 1953; Barboza et al., 2022). It is morphologically distinctive with conspicuous pubescence, primarily purple flowers and fruits with large blackish-brown seeds. The fruits are hot fleshy berries of variable shapes, sizes, and colors (Barboza et al., 2022). *Capsicum pubescens* is the least studied and exploited among domesticated chile species, most likely because of its specific environmental requirements and the high fruit fleshiness, which makes them prone to fast rotting (Eshbaugh, 1993). Its cultivation outside the Americas is infrequent, although it has been introduced and grown as far away as Indonesia (Yamamoto et al., 2013). In recent years, locoto chile market demands have grown due to increased gastronomic and phytochemical interest (Meckelmann et al., 2015; Leyva-Ovalle et al., 2018).

In contrast to the other four domesticated *Capsicum* species (i.e., *C. annuum* L., *C. baccatum* L., *C. chinense* Jacq., *C. frutescens* L.), *C. pubescens* is known only as a cultigen and no ancestral wild population has been found so far (Barboza et al., 2022). Its domestication has been hypothesized to have taken place around 6,000 years ago in Bolivia and/or Peru (DeWitt and Bosland, 2009), followed by a human-assisted range expansion to other areas of the continent, including Central America and Mexico (Heiser and Smith, 1953; Barboza et al., 2022). Despite various attempts to unravel its evolutionary history (e.g., Eshbaugh, 1979; Moscone et al., 2007; Perry et al., 2007; Carrizo García et al., 2016), the origin of *C. pubescens* remains unknown and its evolutionary affinities are controversial. The locoto chile was traditionally placed in the so-called purple-flowered group of *Capsicum* together with two wild chile species, *C. cardenasii* Heiser & P.G.Sm. and *C. eximium* Hunz. (including *C. eshbaughii* Barboza, formerly *C. eximium* var. *tomentosum* Eshbaugh & P.G.Sm.), hypothesis supported by morphological, chemical and cross-breeding data (Heiser and Smith, 1958; Ballard et al., 1970; Eshbaugh and Smith, 1971; Eshbaugh, 1979). These wild chile species, popularly known as ‘ulupicas’, are used locally as hot spices, either cultivated on small farms or harvested directly from the wild (van Zonneveld et al., 2015; Barboza et al., 2022). *Capsicum eximium* and *C. cardenasii* are native to the Central Andes, from center-western Bolivia to north-western Argentina (Barboza et al., 2022). Geographically, part of the cultivation range of *C. pubescens*, and one of the areas proposed as its hypothetical center of origin, overlap with the distribution ranges of these two wild species. Based on the combined evidence, *C. eximium* and *C. cardenasii* have long been suggested as the putative wild progenitors of *C. pubescens* (Pickersgill, 1971; Eshbaugh, 1975, Eshbaugh, 1979), an early hypothesis that was generally accepted but never rigorously tested.

Previous phylogenetic analyses using a wide arrange of molecular markers have attempted to resolve the relationships of all five domesticated chile species and to identify their closest wild relatives (Carrizo García et al., 2022, and references herein). In comparison to the other four cultivated chile species, the phylogenetic position and affinities of *C. pubescens* have not been fully resolved, with the evidence suggesting that *C. pubescens* was either closely related/sister to *C. eximium* and *C. cardenasii* (McLeod et al., 1979, 1983; Choong, 1998; Ince et al., 2010; Ibiza et al., 2012) or, more frequently, recovered as an isolated lineage (Walsh and Hoot, 2001; Ryzhova and Kochieva, 2004; Carrizo García et al., 2016, 2020; Silvar and García-González, 2016; Barboza et al., 2019, 2020). Thus, the informal purple-flowered group s.l. has repeatedly been inferred as paraphyletic. The phylogenetic affinities of *C. pubescens* were most recently addressed in a phylogenetic study of relationships within the genus *Capsicum* based on genome-wide SNP data of 1–3 accessions of each of 36 of its 43 currently recognized species (Carrizo García et al., 2022). This analysis placed *Capsicum pubescens* as a sister species to a small clade encompassing *C. eximium*, *C. eshbaughii* and *C. cardenasii*, with all four species forming the so-called clade Pubescens (Carrizo García et al., 2022). This evidence allowed to narrow down the closest relatives of *C. pubescens*, but the sampling of genetic diversity of the clade Pubescens was insufficient to conclusively infer the nature of the relationships among these four species.

Although ancestor-descendant relationships have been proposed within the clade Pubescens, they have never been resolved, whereas the occurrence of natural hybrids has been repeatedly reported in the group (Eshbaugh, 1975, 1979; Onus and Pickersgill, 2004; Scaldaferro, 2019; Barboza et al., 2022). The impact of recent hybridization and introgression, versus non-contemporary processes like ancient introgression or incomplete lineage sorting (deep coalescence; Twyford and Ennos, 2012), are therefore still unclear. Similarly, the extent (if any) of genetic contribution from wild species *C. cardenasii*, *C. eximium* and *C. eshbaughii* to the genetic variation of cultivated *C. pubescens*, as well as the extent of the *C. pubescens* gene pool, remains largely unknown. Previous phylogenetic studies included a low number of samples per species, thus limiting the power of the phylogenetic inferences. Analyses based on a broader sampling, addressing both genetic variation and geographic distribution of the target species, are thus necessary to shed light on the origin and evolutionary affinities of *C. pubescens*. Over the past decade, the availability of extensive genomic data and development of computational analytical approaches has allowed for the detection of independent lineages with high level of objectivity and statistical rigor. Restriction site-associated DNA sequencing (RADseq; Baird et al., 2008) is a reduced representation sequencing approach that covers a subset of noncoding and coding regions across the entire genome. Frequently used for genomic diversity scans of closely related groups within species or genera (Davey and Blaxter, 2011; Andrews et al., 2016), RADseq has been valuable for delimiting species, reconstructing phylogenies, and inferring evolutionary histories of various plant and animal groups, including the genus *Capsicum* (Carrizo García et al., 2022). In this study, comprehensive population genetic and phylogenetic analyses of RADseq genome-wide data of multiple accessions of *C. pubescens* and its sister species representing genetic and geographical variation of the group were performed to: (1) test existing hypotheses on the phylogenetic relationships of *C. pubescens* and its closest wild relatives, (2) identify patterns of genetic relatedness and structure across this species group, and (3) gain novel insights into the origin and evolutionary history of *C. pubescens*.

## 2 Materials and methods

### 2.1 Sampling

**Figure 1:**
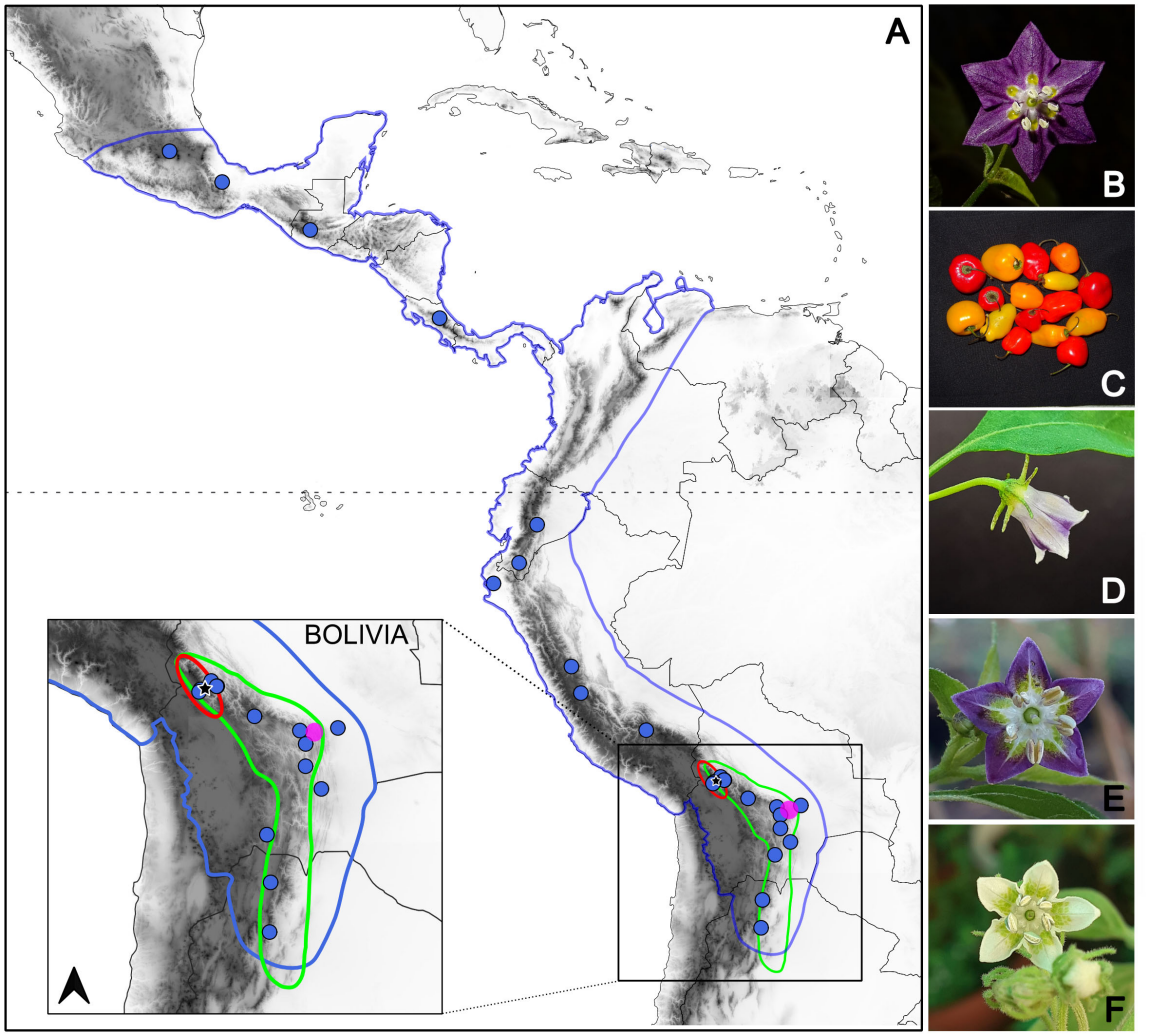
Geographic distribution and morphological characters of analyzed *Capsicum species*. **(A)** Area of *C. pubescens* cultivation in Central-South America (blue) with the details of the native ranges of distribution of *C. eximium* (green), *C. cardenasii* (red), and *C. eshbaughii* (fuchsia) in the inset. Circles on the map indicate the provenance of the *C. pubescens* accessions analyzed. The star points to the city of La Paz, Bolivia. **(B-F)** Flowers of *C. pubescens* **(B)**, *C. cardenasii* **(D)**, *C. eximium* **(E)**, *C. eshbaughii* **(F)** and fruits of *C. pubescens* **(C)**. Photos by NEP and CCG.

A total of 48 samples of *C. pubescens*, *C. cardenasii*, *C. eximium* and *C. eshbaughii* (**Figure 1**), all four species representing the clade Pubescens [Carrizo García et al. (2022); **Supplementary Table 1**] were included in the analyses. One individual of *C. tovarii* Eshbaugh, P.G.Sm. & Nickrent was used as outgroup. The samples were collected across the known distribution ranges of the species (**Figure 1**), except for the cultivated *C. pubescens*, for which 26 accessions were sampled representing the main genetic clusters described in Palombo and Carrizo García (2022). This approach aimed to attempt to cover the whole cultivation range and the genetic variation present in the species. Three individuals identified as artificial hybrids (Barboza et al., 2022) were also included in the analyses (**Supplementary Table 1**). Plant material was collected either from the wild or from plants grown at the Instituto Multidisciplinario de Biología Vegetal (IMBIV, Cordoba, Argentina) and the Botanical Garden of the University of Vienna (HBV, Vienna, Austria).

### 2.2 RADseq library preparation and loci assembly

Genomic DNA was isolated using the DNeasy Plant Mini^®^ kit (Qiagen, Germantown, MD, USA) from leaves dried in silica gel and RADseq libraries were prepared as described in Palombo and Carrizo García (2022). Raw data quality was assessed with FastQC v.0.11.9 (Andrews, 2010). Raw reads were demultiplexed using illumina2bam (https://github.com/gq1/illumina2bam) and process_radtags in Stacks v.2.41 (Catchen et al., 2013) with simultaneous sequence quality filtering (minimum Phred scores set to 20 and allowing a single mismatch in the barcodes).

RADseq loci from all samples were filtered and assembled *de novo* in ipyrad v.0.9.87 (Eaton and Overcast, 2020) using default parameters for diploids except for parameters number 14 (*clust_threshold*) and number 21 (*min_sample_locus*). The threshold for clustering reads within and between individuals was set to 0.88 based on previous results (Carrizo García et al., 2022; Palombo and Carrizo García, 2022). Assemblies with different minimum amounts of samples per locus (*min_sample_locus* 12, 24 and 37) were filtered out to assess the effects of the number of loci and missing data on genomic analysis, i.e., 75% (designated as *min25*), 50% (*min50*) and 25% (*min75*) of missing data, respectively. Given that some analyses are more sensitive to missing data (e.g., population structure analysis, SNAPP; see below), additional filtering was performed, including only biallelic sites with a minor allele frequency above 0.05 using VCFtools v.0.1.16 (Danecek et al., 2011) and pruning to one SNP per locus with the vcf_parser.py script (https://github.com/CoBiG2/RAD_Tools/blob/master/vcf_parser.py). These assemblies were referred to as *filtered*.

### 2.3 Genetic structure analyses

Genetic structure was first analyzed with fineRADstructure v.0.3.3 (Malinsky et al., 2018) using the *.alleles file from the ipyrad output (all SNPs per locus). The allele data was converted using the finerad_input.py script (http://github.com/edgardomortiz/fineRADstructure-tools). fineRADstructure was run following the software pipeline with the default settings and using the associated R script to plot the heatmaps in R v.4.0.3 (R Core Team, 2022). The *min50* dataset (minimal 24 samples per locus, 37,970 loci, 50% missing data) was selected for further analyses because it yielded the best resolution.

The genetic structure was also inferred by applying the sNMF function within the R package LEA v.2.0 (Frichot and François, 2015) to better visualize genomic variation and admixture among individuals. This function calculates ancestry proportions of *K* ancestral populations with least-square estimates. Because these analyses are less tolerant to missing data and rare alleles, the *min75_filtered* dataset (minimal 37 samples per locus, 12,414 unlinked SNPs, 25% missing data) was selected to execute the analysis. sNMF was run for *K*= 1–10, with 100 repetitions, regularization parameter set to 250 and 25% of the genotypes masked to compute the cross-entropy criterion. Bar plots showing ancestry coefficients were obtained using the software R script.

### 2.4 Phylogenetic and species tree inferences

A maximum likelihood tree of all individuals was inferred using IQ-TREE 1.6.12 (Nguyen et al., 2015) in the program web server (Trifinopoulos et al., 2016). The analyses were run with and without the seven putative hybrid individuals detected in the populational analysis. The *min50* dataset (minimal 24 samples per locus, 37,970 loci, 50% missing data) was selected for the analysis because it balanced the number of loci and missing data. Variable sites were filtered out from the *.usnps.phy ipyrad files producing output alignments that could then be used with the ascertainment bias correction (ASC) model. The best nucleotide substitution model was chosen *a priori* using ModelFinder (Kalyaanamoorthy et al., 2017). Node supports were calculated with 1,000 iterations of UltraFast-Bootstrap (UFBoot; Hoang et al., 2018).

A species tree under the multi-species coalescent model was inferred using the SVDquartets algorithm (Chifman and Kubatko, 2014) implemented in PAUP* v4.0a (Swofford, 2003). The *min75_filtered* dataset (9,243 unlinked biallelic SNPs) was used to run the analyses both with and without the seven putative hybrid samples identified in the population analysis. All specimens were treated as independent samples and the “distribute” option for heterozygous sites was applied. All possible quartets were analyzed using the QFM algorithm and node support was assessed by performing 1,000 bootstrap replicates (BS). The IQ-TREE and SVDquartets consensus trees were calculated and node support values were annotated and visualized in FigTree v1.4.3 (Rambaut, 2012). UFBoot ≥ 95% and BS ≥ 80% were considered as strong support. All inferred trees were rooted with *C. tovarii* as outgroup.

The Bayesian coalescent-based approach implemented in SNAPP (Bryant et al., 2012) was applied to infer a species/population tree within the clade Pubescens. A subset of the taxonomic sampling that maximized the number of available SNPs was selected because SNAPP does not incorporate missing data. To that end, the *min75_filtered* dataset was pruned to 19 individuals that represented the main lineages inferred by the clustering and phylogenetic analyses, mostly corresponding to species, except for *C. pubescens*, for which three main groups/lineages were resolved and treated independently in populations’ tree inference. Only sites with no missing data were allowed using VCFtools (1,059 unlinked SNPs in total). The vcf2phylip.py script (https://github.com/edgardomortiz/vcf2phylip) was then used to generate the nexus input file for SNAPP. BEAUti 2 (Bouckaert et al., 2019) was used to create.xml files in which the samples were clustered into species/populations. Two chains, 2 million generations each, logging every 1,000 with the first 10% discarded as burn-in, were run in BEAST 2.6 (Bouckaert et al., 2019) on the CIPRES platform (Miller et al., 2010). The mutation rates (*u* and *v*) were sampled from within the MCMC. Effective chain convergence and effective sample sizes across parameters (ESS ≥ 200) were assessed in Tracer v1.7.1 (Rambaut et al., 2018). The tree files (10% burn-in) were combined and the resulting trees were annotated with LogCombiner v2.6.3 and TreeAnnotator v2.6.3 (Drummond and Rambaut, 2007), respectively. The posterior distribution of all the trees combined was visualized as a cloudogram using DensiTree v.2.2.3 (Bouckaert, 2010) and the maximum clade credibility tree was visualized and annotated in FigTree. Posterior probabilities (PP) ≥ 0.95 were considered as strong support.

### 2.5 Hybridization detection

The extent of hybridization among taxa was first assessed using TreeMix (Pickrell and Pritchard, 2012), a method for inferring the patterns of population splits and reticulation in the history of a set of populations based on the allele frequency data of the whole genome. Samples were grouped into populations by generating *.treemix input files with the populations program in Stacks v.2.41 (Catchen et al., 2013) using the *min75_filtered* dataset, representing genetic clusters/clades as recovered in the structure and phylogenetic analyses. The analysis was run in TreeMix v.1.13 with *C. tovarii* as outgroup. The number of migration events (*m*) was sequentially increased and changes in likelihood with each event added were examined. Obtained trees were plotted in R. The TreeMix subprogram ‘threepop’ was used to calculate *F*3 statistics between populations (Reich et al., 2009) in order to see if admixture was supported.

To further investigate the incidence of hybridization, the potential hybrids and parental taxa were tested with HyDe v.0.4.3 (Blischak et al., 2018), an approach similar to the ABBA-BABA test that uses phylogenetic invariants arising under a coalescent model with hybridization to detect and assign the probability of hybridization both at species/population and individual levels. HyDe tests all possible combinations of input taxa as putative hybrids and parents (P1 and P2) and the parameter γ estimates the genomic contributions of the parents to the hybrid. For this analysis, the *min75* dataset with *C. tovarii* as outgroup was set as input data using the *usnps.phy file from ipyrad. The population map defined all parental individuals as species (*C. cardenasii*, *C. eximium* and *C. pubescens*), and each putative hybrid individual was assigned its own group (i.e., ‘Hybrid’). A file of 21 triplets was created to test the seven putative hybrid individuals as hybrids of the parental species, as suggested by the results of previous analyses.

## 3 Results

### 3.1 Sequencing and SNP loci assembly

A total of 90,534,731 RADseq reads were generated for the 49 analyzed samples. An average of 1,705,693 (± 976,049 SD) high-quality reads per individual were obtained after demultiplexing and filtering (**Supplementary Table 1**). The ipyrad pipeline was run separately for datasets used in downstream analyses: 70,304 loci/316,506 SNPs were retained in the *min25* dataset (**Supplementary Data Sheet S1**), 37,970 loci/184,444 SNPs in the *min50* dataset (**Supplementary Data Sheet S2**), and 13,226 loci/63,355 SNPs in the *min75* dataset (**Supplementary Data Sheet S3**) (75%, 50%, and 25% missing data, respectively). The results obtained from the different assemblies were largely consistent, thus datasets *min50* and *min75* were selected for further analysis.

### 3.2 Genetic structure

**Figure 2:**
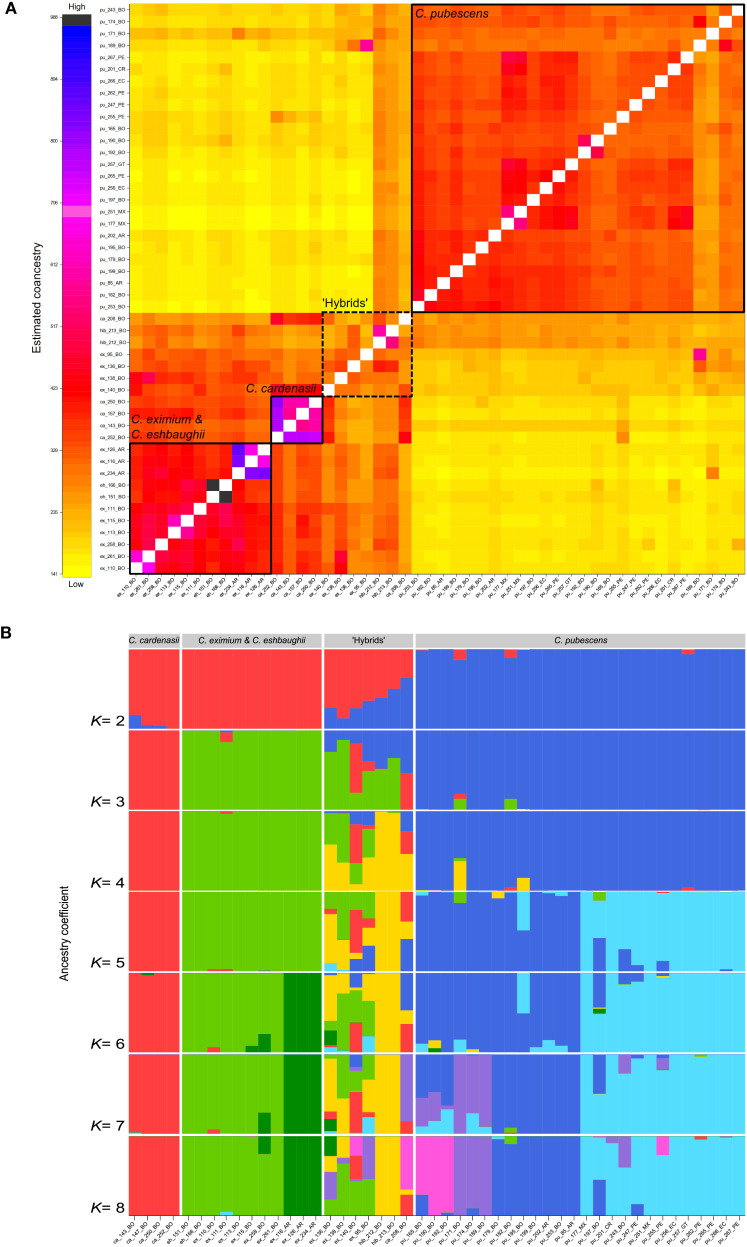
Genetic structure of *C. pubescens* and its sister species with samples names as in **Supplementary Table 1**; tip names indicate sample ID and geographic provenance indicated by the two-letter country code. **(A)** Heatmap plot obtained with fineRADstructure, showing the variation in pairwise co-ancestry among individuals according to the scale shown on the left. Solid squares represent samples from the same species and the dotted squares samples identified as putative hybrids. **(B)** Result of the sNMF analysis for *K*= 2–8. Each bar represents a sample and the colors represent the partitioning of the sample genotype in each group. The samples are sorted by species, except for hybrid individuals.

Two to three main supported clusters were recovered in fineRADstructure analysis indicating a clear separation of *C. pubescens* from the three wild species (*C. cardenasii, C. eximium*, and *C. eshbaughii*) and a weak structuring within *C. pubescens* (**Figure 2A**). The cluster formed by the wild species was genetically more heterogeneous and divided into two subclusters: *C. cardenasii* and *C. eximium* together with *C. eshbaughii*. *Capsicum eshbaughii* accessions were intermingled with *C. eximium* accessions. The individuals labelled as artificial hybrids (ex_136, hib_212, hib_213) were recovered in an intermediate position between the two main clusters of species, together with four samples previously labelled as pure *C. eximium* (ex_95, ex_138, ex_140) and *C. cardenasii* (ca_208). These seven samples showed intermediate co-ancestry values in the heatmap (**Figure 2A**) compared to individuals of the two main clusters, indicating reticulation events.

sNMF structure analysis also revealed the presence of two to three main clusters (**Figure 2B**; **Supplementary Figure 1**), indicative of the genetic differentiation of species and confirmed the admixed composition of the seven samples detected as hybrids in the fineRADstructure analysis. At *K*= 2, the groupings corresponded to *C. cardenasii + C. eximium + C. eshbaughii*, and *C. pubescens*. The most likely model, i.e., *K*= 3, supported three species-structured clusters, with the seven hybrids partially assigned to each group. The first cluster corresponded to *C. cardenasii*, the second was composed of *C. eximium* + *C. eshbaughii*, and the third cluster comprised *C. pubescens* accessions. At *K*= 4, two individuals previously labelled as *C. pubescens* × *C. eximium* hybrids (hib_212, hib_123) were recognized as a separate group. Results of *K*= 5-8 sub-optimal models were also informative, showing a sub-structure within *C. pubescens* (*K*= 5, 7, 8) and *C. eximium* + *C. eshbaughii* (*K*= 6) clusters. At *K*= 5, the two groups of *C. pubescens* samples were recovered corresponding to into individuals from Bolivia and Argentina, and individuals from Peru to Mexico, respectively. Similarly, at *K*= 6, a sub-structure was observed within *C. eximium*, in which the Argentinian samples and the Bolivian samples (plus *C. eshbaughii*) formed two separate groups. For *K*= 7-8, two new subgroups were recognized within *C. pubescens* accessions corresponding to samples collected/marketed in central-western Bolivia (in the surroundings of La Paz city and the town of Villa Serrano). In these models, three samples marketed in La Paz (pu_197, pu_243) and Cusco (pu_255) showed high levels of genetic admixture. The other hybrid samples (ex_95, ex_136, ex_140, ca_208) showed high levels of mixed ancestry with *C. pubescens* from central-western Bolivia contributing to genetic makeup of these individuals.

### 3.3 Phylogenetic relationships and species/population tree estimation

**Figure 3:**
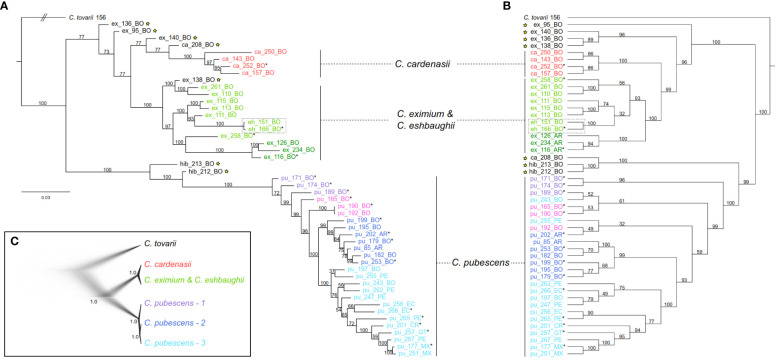
Phylogenetic affinities of *C. pubescens* and its sister species. Samples names as in **Supplementary Table 1**; tip names indicate sample ID and geographic provenance indicated by the two-letter country code; tip colors represent the main genetic clusters resolved by sNMF (**Figure 2B**). **(A)** Best-scoring Maximum Likelihood phylogenetic tree inferred in IQ-TREE with support values next to the branches indicating ultrafast bootstrap (UFBoot). Bar indicates substitutions/site (note that the branch for the outgroup is truncated for graphical reasons). **(B)** The coalescent-based species tree inferred in SVDquartets with bootstrap support (BS) values next to the branches Samples marked with stars represent putative hybrids and samples marked with asterisks were also included in the SNAPP analysis. The dotted square indicates *C. eshbaughii* samples. **(C)** Species trees after SNAPP analysis depicted as cloudogram and consensus tree. Clades and colors are marked in the same in **(A, B)**. Nodal support values are provided as posterior probabilities (PP).

Phylogenetic reconstructions using both the IQ-TREE and SVDquartets approaches were highly congruent (**Figures 3A, B**). Two major clades were resolved with high support: (1) clade of the wild species *C. cardenasii*, *C. eximium* and *C. eshbaughii*, and (2) clade of the domesticated *C. pubescens*. The three artificial hybrid individuals (ex_136, hib_212, hib_213) as well as the four samples identified as putative hybrids in the genetic structure analyses (ex_95, ex_138, ex_140, ca_208) were recovered in intermediate positions between the main clades, except for the sample ex_138 in the IQ-TREE outcome (**Figure 3A**). Excluding artificial and putative hybrids, *C. pubescens* was resolved as sister to a clade consisting of two well-defined subclades of the three other species (**Supplementary Figure 2**). One subclade was represented by *C. cardenasii*, and the other included *C. eximium* and *C. eshbaughii*. As the two samples of *C. eshbaughii* were nested within *C. eximium*, these two species were treated as a single group in the SNAPP and TreeMix analyses (see below). Internal branch supports were mostly moderate-strong (UFBoot= 93-100) throughout the *C. eximium-C. eshbaughii* assemblage, and moderate-weak (BS= 32-74) in some branches in the SVDquartets analysis, linked to alternative topologies (**Figures 3A, B**; **Supplementary Figure 2**). In the SVDquartets outcome, the *C. eximium* samples were consistently recovered into two distinct groups, one representing the accessions from Argentina, and the other the accessions from Bolivia plus *C. eshbaughii*, with internal relationships weakly supported. In the IQ-TREE topology, the *C. eximium* accessions from Bolivia were placed in three different groups, with the *C. eshbaughii* samples sister to the same *C. eximium* accessions as in SVDquartets. All 26 *C. pubescens* accessions formed a well-supported monophyletic group with internal branching structure that was moderately resolved and mostly congruent across both phylogenetic reconstructions (**Figures 3A, B**; **Supplementary Figure 2**). The first splitting branches within this *C. pubescens* clade corresponded to samples collected/marketed in central-western Bolivia (in the surroundings of La Paz city and the town of Villa Serrano). The remaining accessions were recovered as two well-supported main clades, one comprising the Argentinian and other central-Bolivian samples (from Cochabamba and Santa Cruz de la Sierra cities to the south), while the other comprised Peruvian, Ecuadorian, and Central American accessions. The internal branch supports of these clades varied from weak to moderate-strong in the IQ-TREE and SVDquartets trees (UFBoot= 54-100; BS= 49-100). Only minor incongruences in the relationships between some of the accessions (i.e., pu_197, pu_243, pu_255) were detected between both approaches.

Based on the outcome of sNMF, IQ-TREE and SVDquartets analyses, 19 individuals were selected and grouped by species/populations to perform the SNAPP analyses (**Figure 3C**). The taxon partitioning followed the results of the genetic structure and phylogenetic analyses. For species tree inference, *C. eximium* and *C. eshbaughii* were merged, and *C. pubescens* was treated as a single partition (**Supplementary Figure 3**). The *C. pubescens* samples were then split into populations corresponding to the three previously inferred main lineages/groups (identified as *C. pubescens* 1, 2 and 3; **Figure 3C**). Finally, each individual was considered as a single entry to assess the consistency of the relationships found (**Supplementary Figure 3**). Species/populations trees inferred with SNAPP were highly congruent and showed the same well-supported relationships (PP= 1) as those inferred from the concatenated RADseq-SNPs (**Figures 3A, B**). Relationships congruent with the IQ-TREE and SVDquartets trees were also recovered when individuals were considered as single entries, with the cloudogram and the superimposed consensus tree graphically depicting some level of uncertainty in the nodes within *C. pubescens* (PP 0.89-0.25; **Supplementary Figure 3**).

### 3.4 Hybridization detection

The TreeMix analysis inferred branching topology consistent with the phylogenetic and species/population tree reconstructions (**Supplementary Figure 4**). The putative hybrids were consistently recovered as sister to *C. cardenasii-C. eximium* and *C. pubescens*, with one migration event from *C. pubescens* towards the hybrids’ group. Removing the hybrids did not affect the branching pattern. When the hybrids were considered as two separate groups, following the topology of the SVDquartets outcome, one group was found to be sister to *C. cardenasii-C. eximium* and the other group to *C. pubescens*. The tree models with no migration (*m*= 0) events explained 94-96% of the variation in relatedness between the populations; however, the addition of two migration events (*m*= 2) explained 99.9% of the variation. Admixture events were supported by *F*3 statistics (**Supplementary Table 2**).

The HyDe analysis (**Table 1**) inferred hybrid origin for six of the seven putative hybrid accessions identified in other analyses. Significant hybridization between the ‘parental’ species (pairs *C. cardenasii - C. pubescens* and *C. eximium - C. pubescens*) was detected at the species level (γ∼0.5; **Table 1A**). Six of the seven putative hybrid individuals (all except for sample ex_138) showed significant levels of hybridization, with γ-values ranging from 0.26 to 0.61, indicating different levels of hybridization across the individuals tested (**Table 1B**). Most of the admixture occurred at intermediate levels (i.e., γ close to 0.4-0.6), indicating recent hybridization events (samples card_208, exim_95, hib_212 and hib_213). Significant high levels of admixture (i.e., γ close to either 0.1 or 0.9) were detected for samples ex_136 and ex_140, suggesting introgression or older hybridization events.

**Table 1:** Hybridization among analyzed *Capsicum* species.

A					
P1	Hybrid	P2	*Z*-score	*p*-value	γ
*C. cardenasii*	Hybrids	*C. eximium*	-6.276	1.000	0.529
*C. cardenasii*	*C. eximium*	Hybrids	0.893	0.186	0.887
Hybrids	*C. cardenasii*	*C. eximium*	-9.900	1.000	-0.112
*C. cardenasii*	Hybrids	*C. pubescens*	6.443	**0.000**	**0.530**
*C. cardenasii*	*C. pubescens*	Hybrids	-0.680	0.752	0.912
Hybrids	*C. cardenasii*	*C. pubescens*	-0.797	0.787	-0.167
*C. pubescens*	*C. eximium*	Hybrids	-1.167	0.878	1.183
*C. eximium*	*C. pubescens*	Hybrids	-1.143	0.873	0.896
*C. eximium*	Hybrids	*C. pubescens*	8.832	**0.000**	**0.533**
B					
**P1**	**Hybrid**	**P2**	***Z*-score**	***p*-value**	**γ**
*C. cardenasii*	ca_208	*C. pubescens*	8.662	**0.000**	**0.467**
*C. eximium*	ca_208	*C. cardenasii*	-3.071	0.999	0.714
*C. pubescens*	ca_208	*C. eximium*	-1.374	0.915	0.509
*C. cardenasii*	ex_136	*C. pubescens*	0.797	0.213	0.859
*C. eximium*	ex_136	*C. cardenasii*	-7.850	1.000	0.436
*C. pubescens*	ex_136	*C. eximium*	3.776	**0.000**	**0.269**
*C. cardenasii*	ex_138	*C. pubescens*	-1.971	0.976	1.542
*C. eximium*	ex_138	*C. cardenasii*	-4.946	1.000	0.337
*C. pubescens*	ex_138	*C. eximium*	0.811	0.209	0.049
*C. cardenasii*	ex_140	*C. pubescens*	1.409	0.079	0.897
*C. eximium*	ex_140	*C. cardenasii*	-3.415	1.000	0.625
*C. pubescens*	ex_140	*C. eximium*	4.757	**0.000**	**0.259**
*C. cardenasii*	ex_95	*C. pubescens*	4.186	**0.000**	**0.581**
*C. eximium*	ex_95	*C. cardenasii*	-7.214	1.000	0.473
*C. pubescens*	ex_95	*C. eximium*	6.647	**0.000**	**0.430**
*C. cardenasii*	hib_212	*C. pubescens*	1.508	0.066	0.783
*C. eximium*	hib_212	*C. cardenasii*	-6.368	1.000	0.404
*C. pubescens*	hib_212	*C. eximium*	12.946	**0.000**	**0.605**
*C. cardenasii*	hib_213	*C. pubescens*	-9.900	1.000	-0.019
*C. eximium*	hib_213	*C. cardenasii*	-3.639	1.000	0.339
*C. pubescens*	hib_213	*C. eximium*	13.060	**0.000**	**0.552**

The HyDe results for three groups of accessions (P1, P2, Hybrid). The two parents (P1, P2) and the Hybrid are shown for each rooted triplet comparison. The *Z*-score, *p*-value, and gamma (γ) values for each test are shown. Significant values are indicated as bold. **(A)** P1 and P2 (*C. cardenasii*, *C. eximium* or *C. pubescens*) and Hybrid (putative hybrid accessions), and **(B)** P1 and P2 (*C. cardenasii*, *C. eximium* or *C. pubescens*) and each of the putative hybrid accessions treated as a single individual.

## 4 Discussion

This study tested old and proposes new hypotheses on the evolution of the domesticated chile *Capsicum pubescens* and its closest wild allies, based on whole-genome SNP data populational and phylogenetic analyses. The analyses of the extended-sampling datasets using different analytical approaches allowed us to (1) evaluate existing hypotheses on the genetic relationships between these species, (2) identify the main lineages associated with diversification under domestication within *C. pubescens*, and (3) detect hybridization events within the clade Pubescens. All these data allowed us to gain a better understanding of the extent of the primary gene pool of *C. pubescens*. This study provides therefore new insights into the evolutionary history of *C. pubescens* and its human-assisted geographic dispersal in the Americas.

The evolutionary affinities of domesticated *Capsicum* species have previously been studied using molecular data (cf. Liu et al., 2023). Such approaches allowed for tracing the origin of domestication and the subsequent differentiation of most domesticated *Capsicum* species (e.g., Kraft et al., 2014; Scaldaferro et al., 2018; Taitano et al., 2019; Tripodi et al., 2021; Liu et al., 2023). *Capsicum pubescens* is a chile pepper widely cultivated in South America for which, however, no wild ancestor is known. Although the most comprehensive and complete phylogenetic study of the genus *Capsicum* has recently been carried out (Carrizo García et al., 2022), the affinities between *C. pubescens* and its closest wild relatives or putative ancestors, i.e., *C. cardenasii*, *C. eximium* and *C. eshbaughii*, were ambiguously resolved over time due to low sample number of these species, leading to inconclusive inferences. The current study is the first to specifically address the evolutionary relationships of *C. pubescens* and its allies. Highly resolved and strongly supported phylogenetic relationships on populational level were inferred by the extensive utilization of genome-wide SNP data, encompassing significantly larger number of markers than previous studies. This comprehensive dataset included multiple samples representing different geographical areas of distribution and a broad spectrum of genetic diversity of the target species. The results supported *C. pubescens* as a distinct lineage, with *C. cardenasii*, *C. eximium* and *C. eshbaughii* as its closest wild relatives. However, none of these three species could be inferred as a direct ancestor of locoto chile. The same patterns of phylogenetic relationships were recovered in all analyses, consistent with the delimitation of the four species within the clade Pubescens proposed recently (Carrizo García et al., 2022). The proposed Pubescens clade circumscription is also in agreement with the traditional informal placement of the four species known as the purple-flowered group of chiles (Pickersgill, 1971; Eshbaugh, 1975).

RADseq data, originally developed for intraspecific phylogeographic studies (Baird et al., 2008; McCormack et al., 2013), allowed to analyses a large number of phylogenetically informative loci/SNPs. The use of more loci and a higher number of closely related individuals enabled a better characterization of phylogeographic variation, particularly within *C. eximium* and *C. pubescens*, the species with wider distribution ranges. *Capsicum eximium* is the most phenotypically variable ‘ulupica’ species with distribution range spanning contrasting ecogeographical areas, from the Yungas rainforest of Bolivia and northern Argentina to the dry valleys of central Bolivia, and a wide altitudinal gradient (ca. 1000-3000 m). The species exhibits a high level of phenotypic variation (e.g., corolla pigmentation), although it has not been examined exhaustively to date (Eshbaugh, 1982; Barboza et al., 2022; pers. obs.). Current results suggest that geographic factors, such as climate and topography, may have played a role in shaping the structure of genetic variation within the species as Argentinian and Bolivian accessions were found to represent different genetic groups/lineages. Additionally, *C. eshbaughii*, an endangered species that can only be found in a very restricted area of south-central Bolivia (Barboza et al., 2022), was recovered within the (Bolivian) *C. eximium* clade. Initially described as a variety, i.e., *C. eximium* var. *tomentosum* (Eshbaugh and Smith, 1971), it was later recognized as a distinct species under the name *C. eshbaughii* (Barboza, 2011), but its species status has recently been questioned (Carrizo García et al., 2020). The current results do not support a recognition of *C. eshbaughii* at specific level without recognizing *C. eximium* as paraphyletic. Thus, the taxonomic status and evolution of the *C. eximium-C. eshbaughii* assemblage needs to be addressed with extended sampling of both taxa. A few studies have analyzed intraspecific genetic variation of other wild *Capsicum* species across their geographical distribution to understand their evolutionary history and diversity (but see Scaldaferro et al., 2023) and this study demonstrates the profits of extended sampling and genome-wide analyses.

*Capsicum pubescens* accessions were recovered in two main geographically structured groups representing south (from Argentina and Bolivia) and north (northwards from Peru to Mexico) populations. A substructuring was revealed within the Bolivian accessions, with a group from central-western Bolivia (La Paz surroundings). Phylogenetically, *C. pubescens* formed a single clade in which three primary lineages were recognized, distributed mostly in (1) central-western Bolivia, (2) central-southern Bolivia to Argentina, and (3) northern part of the continent from Peru to Mexico. The central-western Bolivian sample set was found to have diverged earlier than the other two, but did not consistently form a monophyletic group. The diversification of this lineage within *C. pubescens* is consistent with the geographical patterns of genetic variation reported earlier (Palombo and Carrizo García, 2022), revisited here in an evolutionary framework thus adding a temporal dimension. The central-western Bolivian accessions, characterized by unique genetic variation congruent with distinct plant morphology, including characters like near-absence of pubescence, small mostly 5-merous flowers, and the smallest and fleshiest fruits (**Supplementary Figure 5**), hint at a minor/incomplete domestication syndrome or de-domesticated phenotype (Palombo and Carrizo García, 2022). These plants collected *in situ* from a home garden and disturbed sites exhibited higher genetic diversity than other *C. pubescens* genetic groups (Palombo and Carrizo García, 2022), suggesting that they may more closely resemble the ancestral gene pool of the species. Accessions from the other two sister lineages (i.e., central-southern Bolivia to Argentina, and northwards from Peru to Mexico) displayed typical characteristics of the *C. pubescens* cultigen (**Supplementary Figure 5**). The current results suggest that the locoto chile has diversified from central-western Bolivia towards the south and north of the continent, possibly due to human-assisted germplasm dispersal. Moreover, measures of genetic diversity revealed that the northern samples group exhibits the least diversity (Palombo and Carrizo García, 2022), suggesting the occurrence of a bottleneck or founder effect during the locoto introduction to the north of the continent. This is in agreement with previous reports of the species being introduced to Central America and Mexico in the 20th century, rather than the development of historical cultivars (Heiser and Smith, 1953; Barboza et al., 2022). No research so far has explored in depth the locoto chile domestication and dispersal process, thus, the current data provide a good basis for such studies. Future analyses might profit from more detailed information about cultivation and sales locations of the studied samples for better understanding of intraspecific genetic dynamics.

The Central Andes (i.e., Bolivia, Peru and Ecuador) have been inferred as the ancestral area of origin of the entire clade Pubescens (Carrizo García et al., 2022). The lineage of *C. pubescens* was hypothesized to have diverged in the upper Pliocene, earlier than the clade formed by *C. cardenasii*, *C. eximium* and *C. eshbaughii* that diversified from the mid-Pleistocene (Carrizo García et al., 2022). These inferences imply a long period of evolutionary divergence of the different lineages within the clade Pubescens. Despite of the early origin of the *C. pubescens* lineage, the archaeological record indicates that its domestication, leading to the known extant form of the species, might have only taken place a few millennia ago [6,500 cal. BP (Perry et al., 2007; Chiou et al., 2014)]. The central Bolivian mid-highlands have been proposed as the hypothetical center of origin for *C. pubescens*, which is consistent with a greater morphological variation of locoto chile in this region as well as with the presence of its wild sister species (Eshbaugh, 1979; McLeod et al., 1983; DeWitt and Bosland, 2009). Previous studies have also shown that plants with smaller fruits (more ancestral character) are found in Bolivia, supporting the hypothesis that the Bolivian region would harbor plants most resembling the ancestral gene pool of *C. pubescens* (Eshbaugh, 1979; Palombo and Carrizo García, 2022). However, no archaeological evidence exists to support this hypothesis. It is possible that the wild ancestor of the extant *C. pubescens* may have become extinct. There are a few examples of crop plant species for which no wild ancestral populations have been identified yet, such as greater yam (*D. alata* L.; Chaïr et al., 2016). *Capsicum pubescens* cultivation is mainly restricted to particular environmental conditions, which led to the hypothesis that perhaps the only sites in which the wild forms could have grown have been occupied by humans and their cultigens (Rick, 1950). Subsequent competition and/or hybridization of wild ancestral forms with the “improved” domesticated forms might have led to the loss of the original genetic and morphological diversity among the wild forms, and rendering their identification difficult or impossible (Rick, 1950). The earliest *C. pubescens* domesticates could have also been extracted from the wild prior to the domestication bottleneck and their parental population(s) may have disappeared either as the result of early human activities or spontaneously over time. The current data suggest central-western Bolivia as a potential region in which to search for the origin of *C. pubescens*, specifically in the inter-Andean valleys from the south-east of La Paz to the north-west. Future work in this geographic area will allow for a better understanding of the variability of the cultigen and its geographical distribution, and will in turn allow for better understanding of its origin and diversification.

The combined evidence strongly supports the hypothesis that none of the three closest allied species of *C. pubescens* can unequivocally be identified as its wild progenitor (Carrizo García et al., 2022). No direct ancestor-descendant relationships could be recovered within this group, but, since the genetic diversity of a species can exceed its taxonomic limits, the suggestion that primary (extended) gene pool of *C. pubescens* includes the wild *C. eximium* and *C. cardenasii* (van Zonneveld et al., 2015) is reinforced. The existence of such gene pool, also known as “Pubescens complex”, has received support from cross-breeding experiments between *C. pubescens* and the wilds *C. eximium* and *C. cardenasii* (Eshbaugh, 1979; Tong and Bosland, 1999; Onus and Pickersgill, 2004) and is now statistically supported by the current results. Successful reciprocal crosses of *C. eshbaughii* with both *C. eximium* and *C. cardenasii* (Eshbaugh and Smith, 1971; pers. obs.) suggest that *C. eshbaughii* is also part of this gene pool. Moreover, the current study revealed the presence of natural hybrids between either *C. pubescens* and *C. eximium* or *C. pubescens* and *C. cardenasii*. In many traditional communities across South-Central America, wild *Capsicum* species are often found growing close to domesticated chiles, in home gardens or at the edges of cultivated fields, where they can readily hybridize with the cultigens (van Zonneveld et al., 2015; Pérez-Martínez et al., 2022). Disregarding the two hybrids previously described as experimental crosses between *C. pubescens* and *C. eximium* (Barboza et al., 2022), the remaining putative hybrids were inferred to represent hybridization events, with a genetic contribution of the *C. pubescens* cluster from central-western Bolivia (surroundings of La Paz). Some of these hybrids were collected from or near home gardens (Barboza et al., 2022; unpublished notes) indicating that cross-breeding between cultigens and wild relatives does occur. Two hybrid samples were collected in the area of dry valleys of Luribay (ca. 150 km south-east of La Paz, Bolivia) where *C. cardenasii* and *C. eximium* grow (Barboza et al., 2022), further suggesting that the species may freely hybridize in sympatry, as was also observed earlier (Eshbaugh, 1975, 1979). The Luribay valley is therefore a potential natural laboratory site for the study of hybridization and introgression across the Pubescens clade and their potential evolutionary and/or ecological impact (Twydord and Ennos, 2012; Taylor and Larson, 2019). Although there is no direct evidence of wild-to-domesticated species transition between the current members of the clade Pubescens, gene flow between its species would impact the extent and maintenance of present genetic variation of *C. pubescens* gene pool. Understanding gene flow between locoto chile and its wild relatives in their native range is crucial for *in situ* conservation of genetic diversity, a globally recognized approach to safeguard plant genetic resources alongside *ex situ* conservation strategies (Hammer et al., 2003; Wambugu and Henry, 2022). This knowledge would allow a design of measures to prevent genetic homogenization and effectively conserve the diversity of *C. pubescens* gene pool.

## 5 Conclusion

Analyses of genome-wide SNP data using population genetic and phylogenetic approaches shed new light on the evolutionary history of the cultivated locoto chile, *C. pubescens*. The results clearly demonstrated that this species forms a monotypic lineage that is sister to a group of three other wild *Capsicum* species from the Central Andes. The analysis of high number of samples representing genetic and geographical variation of the four target species allowed for the detection of hybridization events between these taxa and also for the identification of primary lineages associated with the diversification under domestication of *C. pubescens*. The highlands of central Bolivia, south-east to the north-west of La Paz, were hypothesized to represent the center of origin of *C. pubescens*. More extensive sampling of the populations from this region will allow for more rigorous testing of this hypothesis. The new inferences of evolutionary and phylogenetic relationships under the domestication form new basis for germplasm conservation and breeding strategies, and will also be fundamental for guiding further research of the locoto chile and its wild relatives.

## Data availability statement

The datasets presented in this study can be found in the **Supplementary Material** of this article. The raw data are available in the NCBI Short Read Archive (SRA; BioProject ID PRJNA1048933, BioSample accessions SAMN38671573-SAMN38671621).

## Author contributions

NEP: Conceptualization, Formal Analysis, Investigation, Methodology, Writing – original draft, Writing – review & editing. HW-S: Funding acquisition, Resources, Writing – review & editing. CCG: Conceptualization, Funding acquisition, Methodology, Project administration, Resources, Supervision, Writing – review & editing.
