# Supplementary material for: Evolutionary relationships, hybridization and diversification under domestication of the locoto chile (Capsicum pubescens) and its wild relatives
Source: Front Plant Sci. 2024 Feb 23;15:1353991. doi: 10.3389/fpls.2024.1353991 (PMC10924304; doi:10.3389/fpls.2024.1353991)
Supplement: Supplementary file 4 [file DataSheet_4.docx]

Supplementary Material

# Supplementary Data

**Data S1.** *min25* dataset. RAD-seq SNPs *de novo* alignment of 49 samples of *Capsicum pubescens* and sister species (minimal 12 samples per locus; 75% missing data). FASTA file.

**Data S2.** *min50* dataset. RAD-seq SNPs *de novo* alignment of 49 samples of *Capsicum pubescens* and sister species (minimal 24 samples per locus; 50% missing data). FASTA file.

**Data S3.** *min75* dataset. RAD-seq SNPs *de novo* alignment of 49 samples of *Capsicum pubescens* and sister species (minimal 37 samples per locus; 25% missing data). FASTA file.

# Supplementary Figures and Tables

##
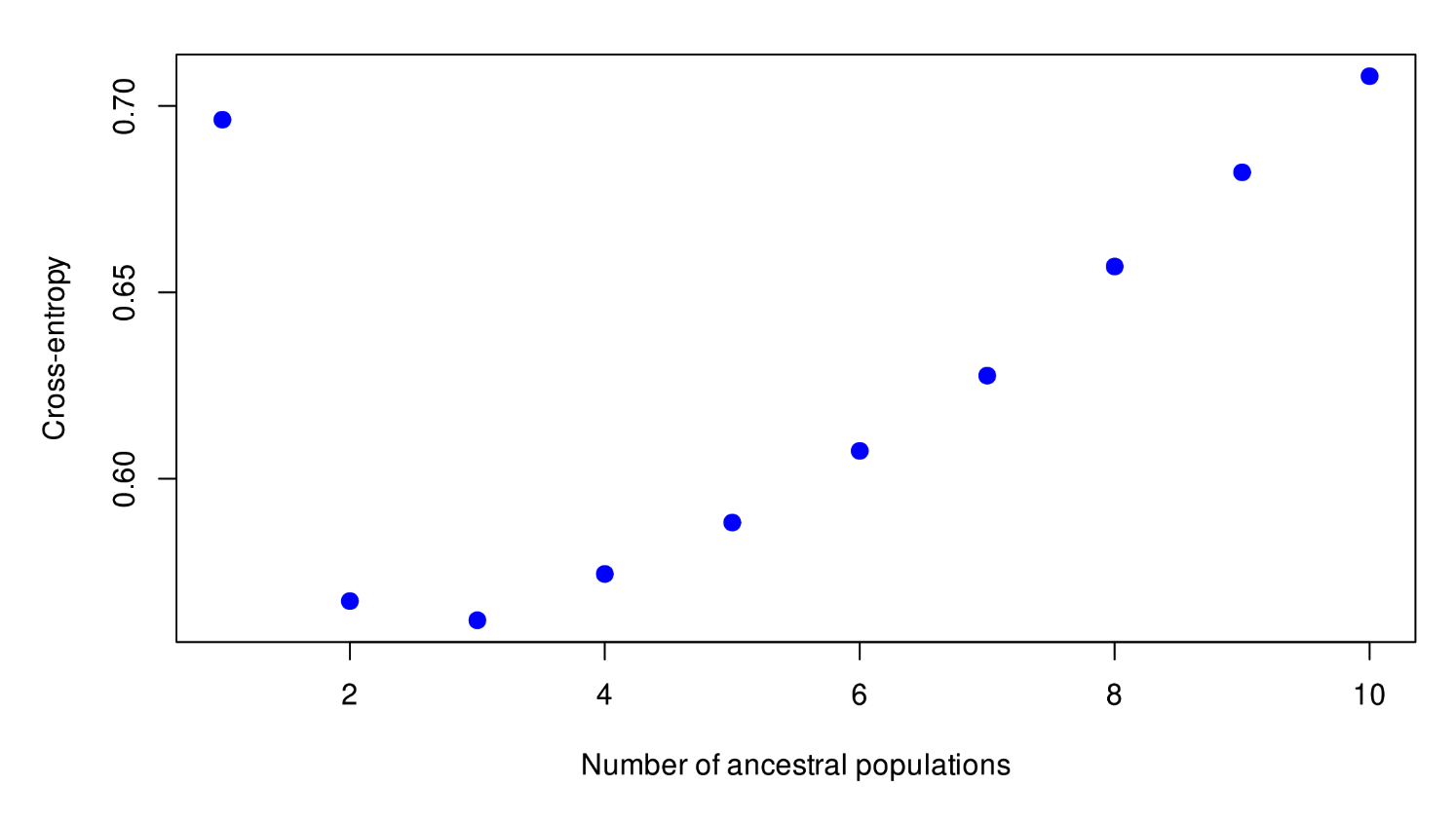
Supplementary Figures

**Supplementary Figure 1.** Cross-entropy values based on sNMF analyses for *K*= 1–10 assumed ancestral genetic groups.

**
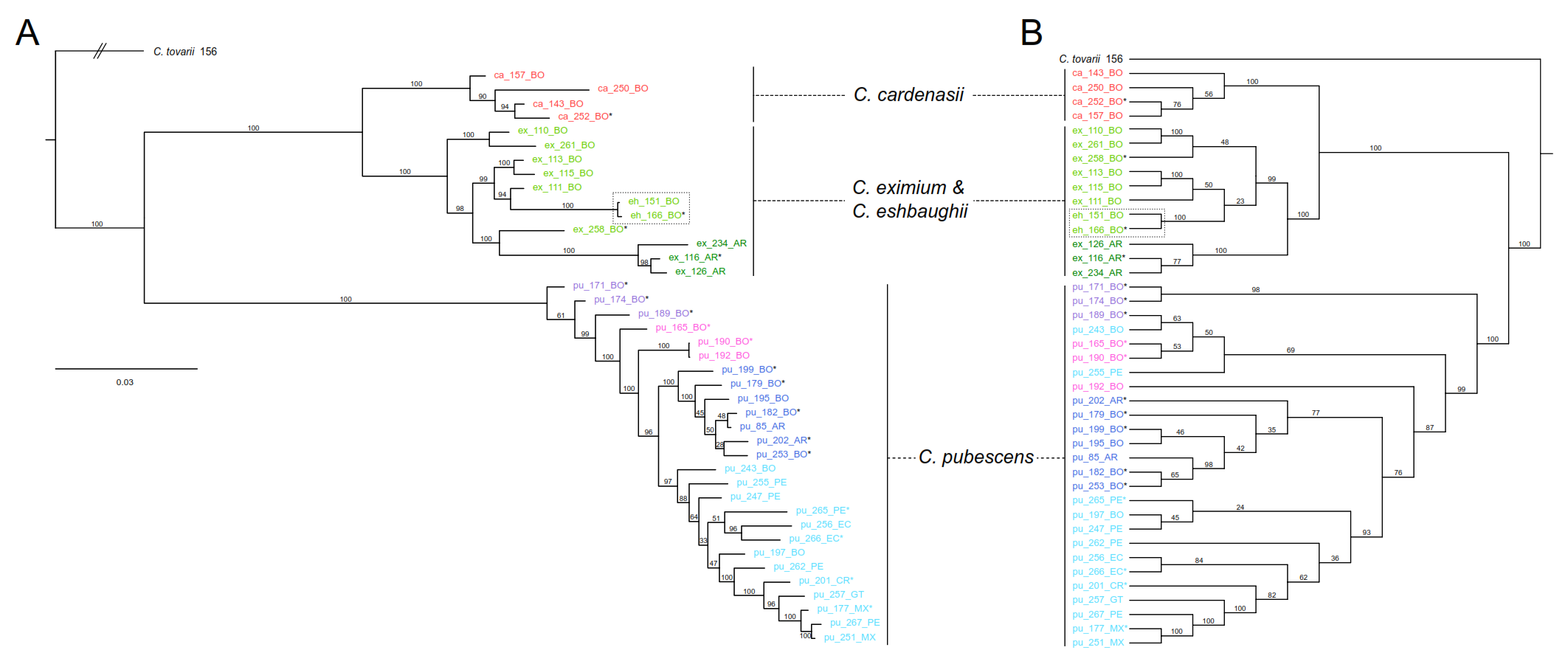
Supplementary Figure 2.** Phylogenetic affinities of *C. pubescens* and its sister species excluding putative hybrid individuals. The names of the samples follow Supplementary Table 1; tip names indicate sample ID and geographic provenance indicated by the two-letter country code; tip colors represent the main genetic clusters resolved by sNMF (Figure 2B)*.* **(A)** Best-scoring Maximum Likelihood phylogenetic tree inferred in IQ-TREE. Support values next to the branches correspond to ultrafast bootstrap (UFBoot). Bar indicates substitutions/site (note that the branch for the outgroup is truncated for graphical reasons). **(B)** The coalescent-based species tree inferred in SVDquartets with bootstrap support (BS) values next to the branches. Samples marked with asterisks correspond to the samples used in the SNAPP analysis.

**
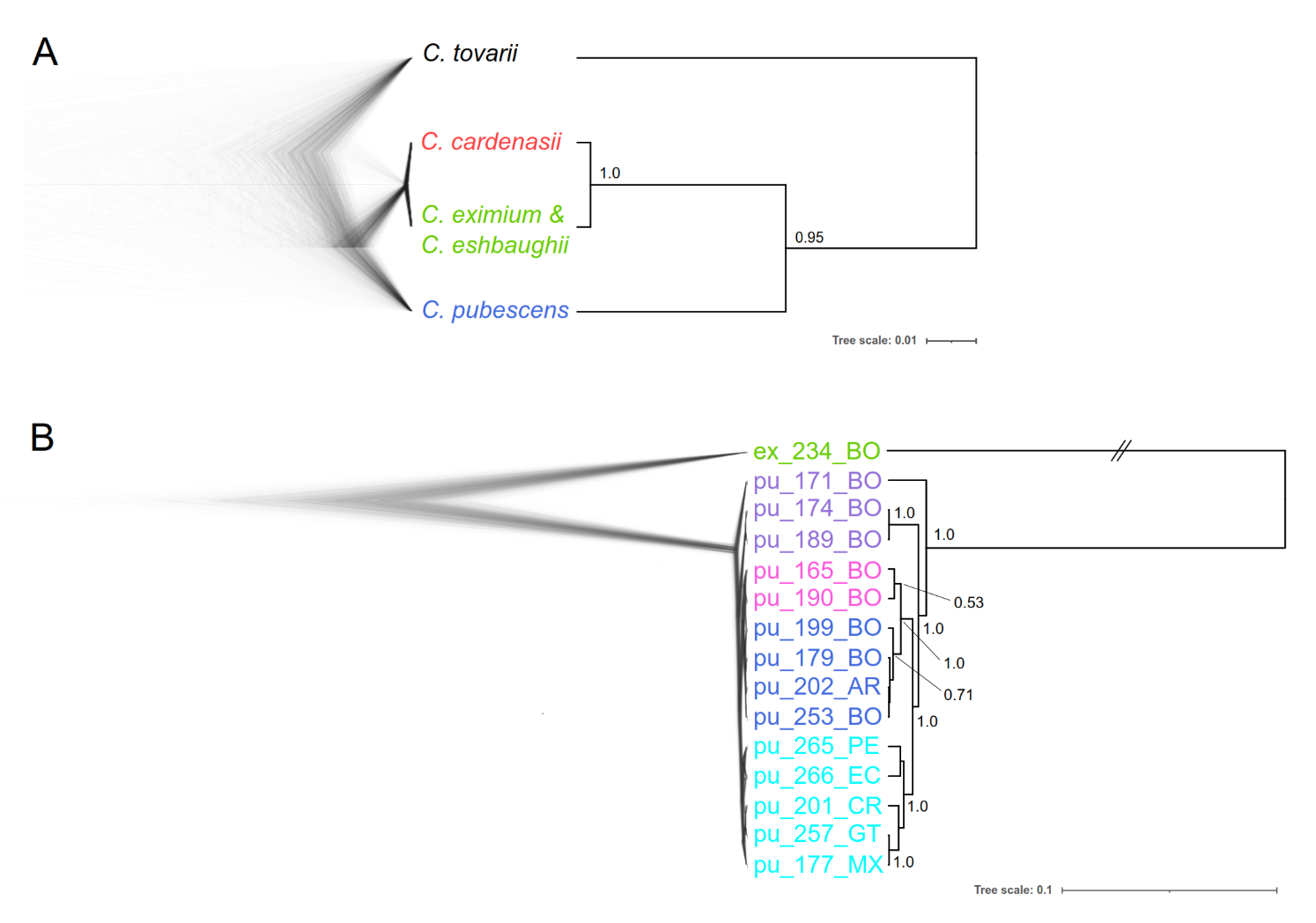
Supplementary Figure 3.** Species trees after SNAPP analysis depicted as cloudograms (left) and consensus trees (right). **(A)** Consensus tree with samples grouped into species. **(B)** Consensus tree with individuals considered as single entries (note that the branch for the outgroup is truncated for graphical reasons). Nodal support values are posterior probabilities (PP). Bar indicates substitutions/site.

**
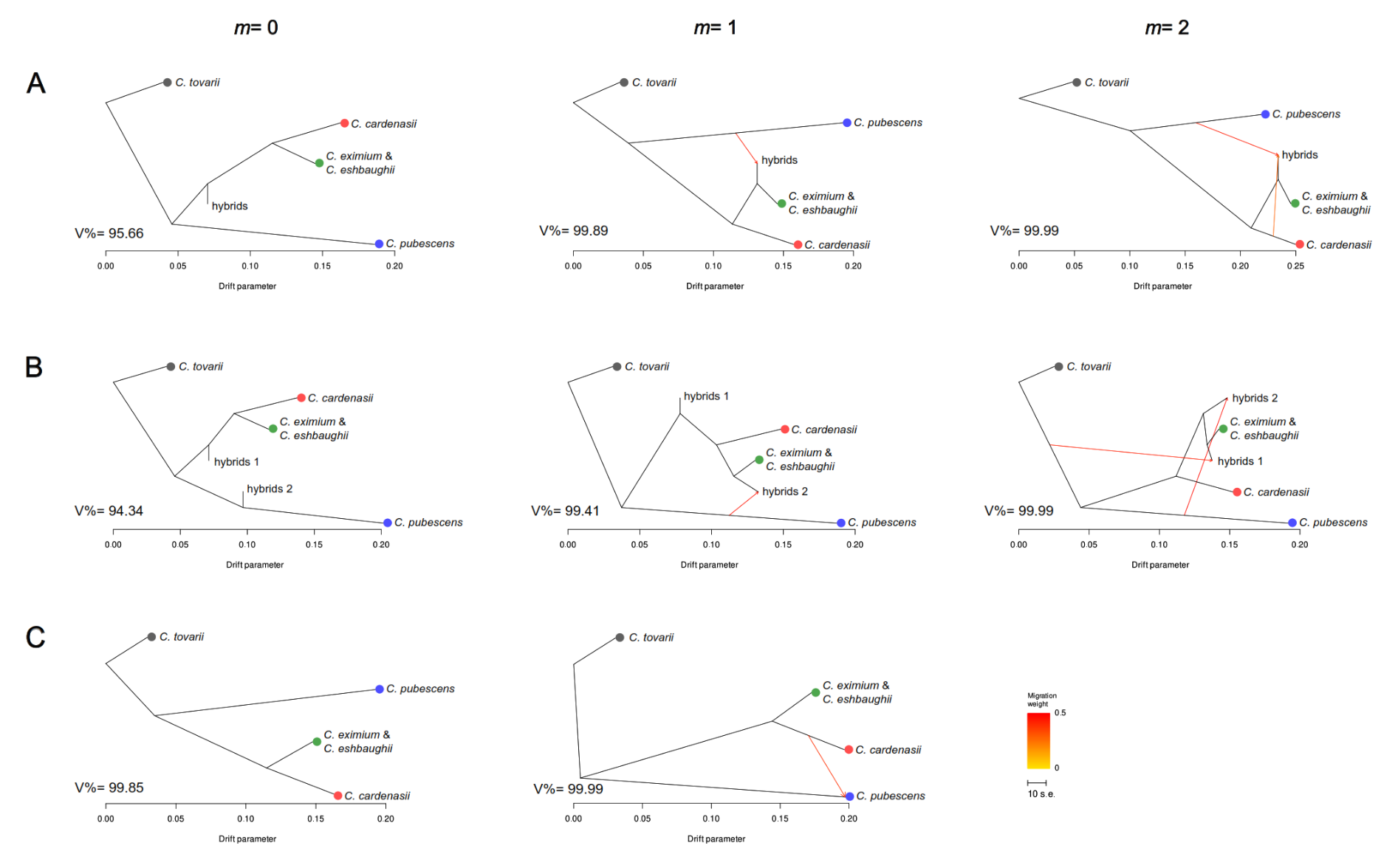
Supplementary Figure 4.** Maximum likelihood trees inferred using TreeMix. **(A)** all putative hybrids treated as a single group (hyb), **(B)** putative hybrids considered as two groups following the SVDquartets outcome, and **(C)** putative hybrids excluded. In all three trees, the arrow is indicative of the direction of gene flow for the models with migration events (i.e., *m* = 0–2). Variance of each model (V%) defined on the left-hand side. All trees were rooted with *C. tovarii*.


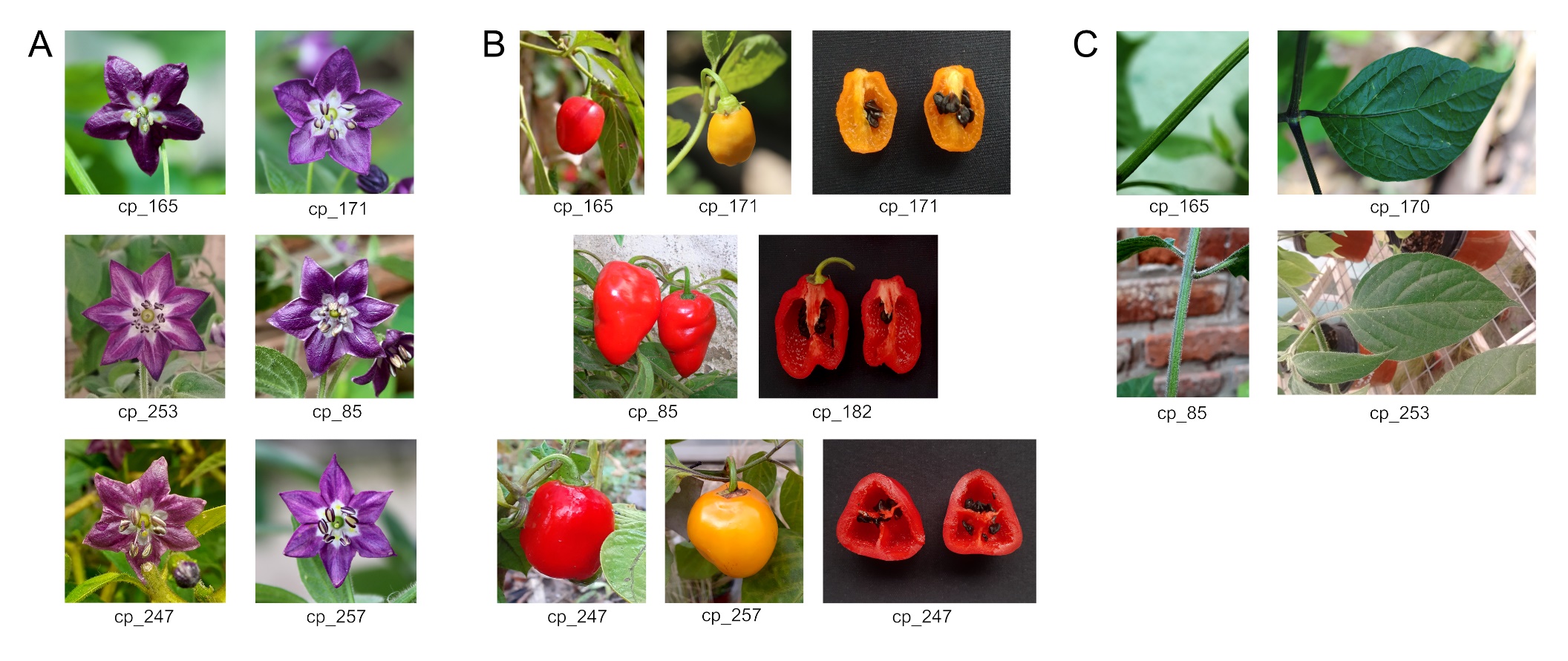
**Supplementary Figure 5.** Morphological variation among the main groups of the studied *C. pubescens* accessions. Representative images showing: **(A)** corolla color and number of petals, **(B)** fruit shape and color, and **(C)** variable levels of pubescence. Accession numbers are indicated as in Supplementary Table 1. Photos by NP and CCG.

## Supplementary Tables

**Supplementary Table 1.** Metadata of the *Capsicum* materials analyzed. Collection data, identification numbers, and number of RADseq reads of each individual. CGN= genebank at the Centre for Genetic Resources, Wageningen University, The Netherlands. LP Co.= Semillas La Palma Company, Germany.

| **ID** | **Taxon** | **Locality** | **Voucher** | **Source** | **Raw reads** | **Filtered reads** |
| --- | --- | --- | --- | --- | --- | --- |
| ca_143_BO | *C. cardenasii* | Bolivia: La Paz, Peña Colorada | Barboza 4884 | wild | 871053 | 861607 |
| ca_157_BO | *C. cardenasii* | Bolivia: La Paz, La Paz | Carrizo García 76 | local market | 789584 | 769979 |
| ca_208_BO | *C. cardenasii* | Bolivia: La Paz, Pucuma | Barboza 4882 | home garden | 1003226 | 912175 |
| ca_250_BO | *C. cardenasii* | Bolivia (unknown) | Carrizo García s.n. | WUR, CGN 20497 | 2622955 | 2397087 |
| ca_252_BO | *C. cardenasii* | Bolivia: La Paz, Sorata | Carrizo García s.n. | wild | 2732617 | 2674567 |
| eh_151_BO | *C. eshbaughii* | Bolivia: Santa Cruz, Samaipata | Carrizo García & Fernández 67 | wild | 1767334 | 1633267 |
| eh_166_BO | *C. eshbaughii* | Bolivia: Santa Cruz, Samaipata | Carrizo García s.n. | wild | 5500840 | 5300826 |
| ex_110_BO | *C. eximium* | Bolivia: Cochabamba, Totora | Carrizo García et al. 93 | wild | 1031107 | 1020444 |
| ex_111_BO | *C. eximium* | Bolivia: Chuquisaca, La Palizada | Carrizo García et al. 31 | wild | 920201 | 905670 |
| ex_113_BO | *C. eximium* | Bolivia: Santa Cruz, Moro Moro | Carrizo García et al. 33 | wild | 833022 | 882576 |
| ex_115_BO | *C. eximium* | Bolivia: Santa Cruz, Vallegrande | Carrizo García et al. 39 | wild | 1319848 | 1301041 |
| ex_116_AR | *C. eximium* | Argentina: Jujuy, El Fuerte | Carrizo García & Fernández 53 | wild | 1017302 | 1006943 |
| ex_126_AR | *C. eximium* | Argentina: Salta | Carrizo García s.n. | wild | 822123 | 808203 |
| ex_136_BO | *C. eximium* × *C. pubescens* | Bolivia: Chuquisaca, Campo Redondo | Barboza 4924 | home garden | 691187 | 670847 |
| ex_138_BO | *C. eximium* | Bolivia: Cochabamba, Totora | Barboza 4896 | home garden | 1894278 | 1761815 |
| ex_140_BO | *C. eximium* | Bolivia: La Paz, Luribay | Barboza 4885 | wild | 2132368 | 1624360 |
| ex_234_AR | *C. eximium* | Argentina: Jujuy, San Francisco | Palombo & Fernández 12 | wild | 4963787 | 4796612 |
| ex_258_BO | *C. eximium* | Bolivia: Chuquisaca, Tomina | Carrizo García s.n. | wild | 2583180 | 2447628 |
| ex_261_BO | *C. eximium* | Bolivia: Cochabamba, Totora | Carrizo García s.n. | wild | 3776201 | 3675521 |
| ex_95_BO | *C. eximium* | Bolivia: Santa Cruz | Barboza 1905 | home garden | 800192 | 781334 |
| hib_212_BO | *C. pubescens* × *C. eximium* | Bolivia: Chuquisaca, Campo Redondo | Barboza 4925 | home garden | 3190019 | 3105402 |
| hib_213_BO | *C. eximium* × *C. pubescens* | Bolivia: Chuquisaca, Campo Redondo | Barboza 4926 | home garden | 2209545 | 2093614 |
| pu_165_BO | *C. pubescens* | Bolivia: La Paz, Apa Apa | Carrizo García 75 | wild | 1381022 | 1261441 |
| pu_171_BO | *C. pubescens* | Bolivia: La Paz, Huancané | Barboza 4889 | wild | 948413 | 885680 |
| pu_174_BO | *C. pubescens* | Bolivia: La Paz, Coroico | Barboza 4890 | home garden | 1899800 | 1817337 |
| pu_177_MX | *C. pubescens* | Mexico: Mexico City, Coyoacán | Chiarini s.n. | local market | 2586734 | 2299246 |
| pu_179_BO | *C. pubescens* | Bolivia: Cochabamba | Carrizo García s.n. | market | 1476390 | 1403666 |
| pu_182_BO | *C. pubescens* | Bolivia: Santa Cruz, Moro Moro | Carrizo García et al. 35 | home garden | 753672 | 713598 |
| pu_189_BO | *C. pubescens* | Bolivia: La Paz, La Paz | Carrizo García s.n. | local market | 1508887 | 1432400 |
| pu_190_BO | *C. pubescens* | Bolivia: Chuquisaca, Villa Serrano | Carrizo García s.n. | local market | 2110489 | 1968839 |
| pu_192_BO | *C. pubescens* | Bolivia: Chuquisaca, Villa Serrano | Carrizo García s.n. | local market | 2126767 | 1982643 |
| pu_195_BO | *C. pubescens* | Bolivia: Santa Cruz, Comarapa | Carrizo García s.n. | local market | 1630556 | 1503309 |
| pu_197_BO | *C. pubescens* | Bolivia: La Paz, La Paz | Carrizo García s.n. | local market | 2362268 | 2041000 |
| pu_199_BO | *C. pubescens* | Bolivia: Potosí, Tupiza | Carrizo García s.n. | local market | 1428175 | 1246406 |
| pu_201_CR | *C. pubescens* | Costa Rica (unknown) | Carrizo García s.n. | LP Co. 'Costa Rican Red' | 1850043 | 1777304 |
| pu_202_AR | *C. pubescens* | Argentina: Jujuy, Humahuaca | Carrizo García s.n. | local market | 2634423 | 2377950 |
| pu_243_BO | *C. pubescens* | Bolivia: La Paz, La Paz | Carrizo García s.n. | local market | 2785501 | 1319629 |
| pu_247_PE | *C. pubescens* | Peru: Piura | Carrizo García s.n. | local market | 1145483 | 1105495 |
| pu_251_MX | *C. pubescens* | Mexico (unknown) | Carrizo García s.n. | LP Co. 'Manzano rojo' | 1010453 | 933189 |
| pu_253_BO | *C. pubescens* | Bolivia: Santa Cruz, Muyupampa | Carrizo García s.n. | local market | 2091852 | 1956892 |
| pu_255_PE | *C. pubescens* | Peru: Cusco, Cusco | Scaldaferro s.n. | local market | 2092730 | 1941007 |
| pu_256_EC | *C. pubescens* | Ecuador: Azuay | Carrizo García s.n. | LP Co. Turbo Pube | 2576762 | 2387979 |
| pu_257_GT | *C. pubescens* | Guatemala: Quiché | Carrizo García s.n. | LP Co. 'CAP 363' | 2585141 | 2431794 |
| pu_262_PE | *C. pubescens* | Peru: Junín | Carrizo García s.n. | WUR, CGN 23768 | 1012055 | 930939 |
| pu_265_PE | *C. pubescens* | Peru: Huánuco | Carrizo García s.n. | WUR, CGN 22108 | 1693733 | 1573667 |
| pu_266_EC | *C. pubescens* | Ecuador: Loja | Carrizo García s.n. | LP Co. 'Ají Largo' | 1126367 | 998700 |
| pu_267_PE | *C. pubescens* | Peru (unknown) | Carrizo García s.n. | LP Co. 'Canario' | 1402337 | 1127357 |
| pu_85_AR | *C. pubescens* | Argentina: Salta, Salta | Carrizo García 28 | local market | 872440 | 858353 |
| to_156 | *C. tovarii* | Peru: Huancavelica | Carrizo García 74 | WUR, CGN 22876 | 1970269 | 1930294 |

**Supplementary Table 2**. Comparative populational analyses for which *F*3 statistics were significantly negative (*Z* score ≤ 3) are reported, indicating that Population 1 contains admixture from Populations 2 and 3. Comparisons in **(A)** ‘hybrids’, **(B)** ‘hybrids 1’-‘hybrids 2’, and **(C)** ‘no hybrids’ correspond to groupings defined in Supplementary Figure 4.

| **A. hybrids** | | | | | | | |
| --- | --- | --- | --- | --- | --- | --- | --- |
| Significant *F*3 Statistics | |  |  | |  | | |
| **Pop (1;2,3)** | ***F*3** | | | **SE** | | ***Z* score** |  |
| hyb; card, pube | -0.0550 | | | 0.0013 | | -43.0405 |  |
| hyb; exim, pube | -0.0719 | | | 0.0010 | | -73.4927 |  |
|  |  | |  | |  | | |
| **B. hybrids 1 and 2** | | | | | | | |
| Significant *F*3 Statistics | | | | | | | |
| **Pop (1;2,3)** | ***F*3** | | | **SE** | | ***Z* score** |  |
| hyb1; card, hyb2 | -0.0065 | | | 0.0012 | | -5.6273 |  |
| hyb1; card, pube | -0.0201 | | | 0.0014 | | -13.9674 |  |
| hyb2; card, pube | -0.0668 | | | 0.0015 | | -44.4684 |  |
| hyb1; exim, hyb2 | -0.0097 | | | 0.0009 | | -10.2153 |  |
| hyb1; exim, pube | -0.0389 | | | 0.0011 | | -35.0314 |  |
| hyb2; hyb1, pube | -0.0533 | | | 0.0013 | | -39.7323 |  |
| hyb2; exim, pube | -0.0825 | | | 0.0012 | | -66.1618 |  |
|  | |  |  | |  | | |
| **C. no hybrids** | | | | | | | |
| No significant *F*3 values recovered | | | | | | | |
